# Specificity Protein 1: A Protein With a Two-Sided Role in Ischemic Stroke

**DOI:** 10.3389/fncel.2021.757670

**Published:** 2021-12-14

**Authors:** Qinyang Yu, Wangyang Liu, Zhuohui Chen, Mengqi Zhang

**Affiliations:** ^1^Department of Neurology, Xiangya Hospital, Central South University, Changsha, China; ^2^National Clinical Research Center for Geriatric Disorders, Xiangya Hospital, Central South University, Changsha, China

**Keywords:** ischemic stroke, oxidative stress, cerebral edema, neuroprotection, specificity protein 1 (SP1)

## Abstract

Stroke is one of the leading causes of death and disability worldwide. However, there is a lack of effective medications to speed up the recovery process. Ischemic stroke, as the result of cerebral infarction or cerebral artery narrowing, is accompanied by hemiplegia or impaired consciousness. There are many transcription factors involved in the development of this condition, whose alterations can influence or signal the prognostic outcomes of ischemic stroke. Among them, the augmented expression of specificity protein 1 (SP1) can participate in the progression of the disease by binding DNA to regulate the transcriptions of many genes. Different studies have provided different answers as to whether SP1 plays a positive or a negative role in ischemic stroke. On the one hand, SP1 can play a cytoprotective role as both an antioxidant and anti-apoptotic agent for neurons and glial cells. On the other hand, it can also damage neuronal cells by promoting inflammation and exacerbating brain edema. In this review, we highlight the roles of SP1 in ischemic stroke and shed light on the underlying mechanism.

## Introduction

**Figure 1:**
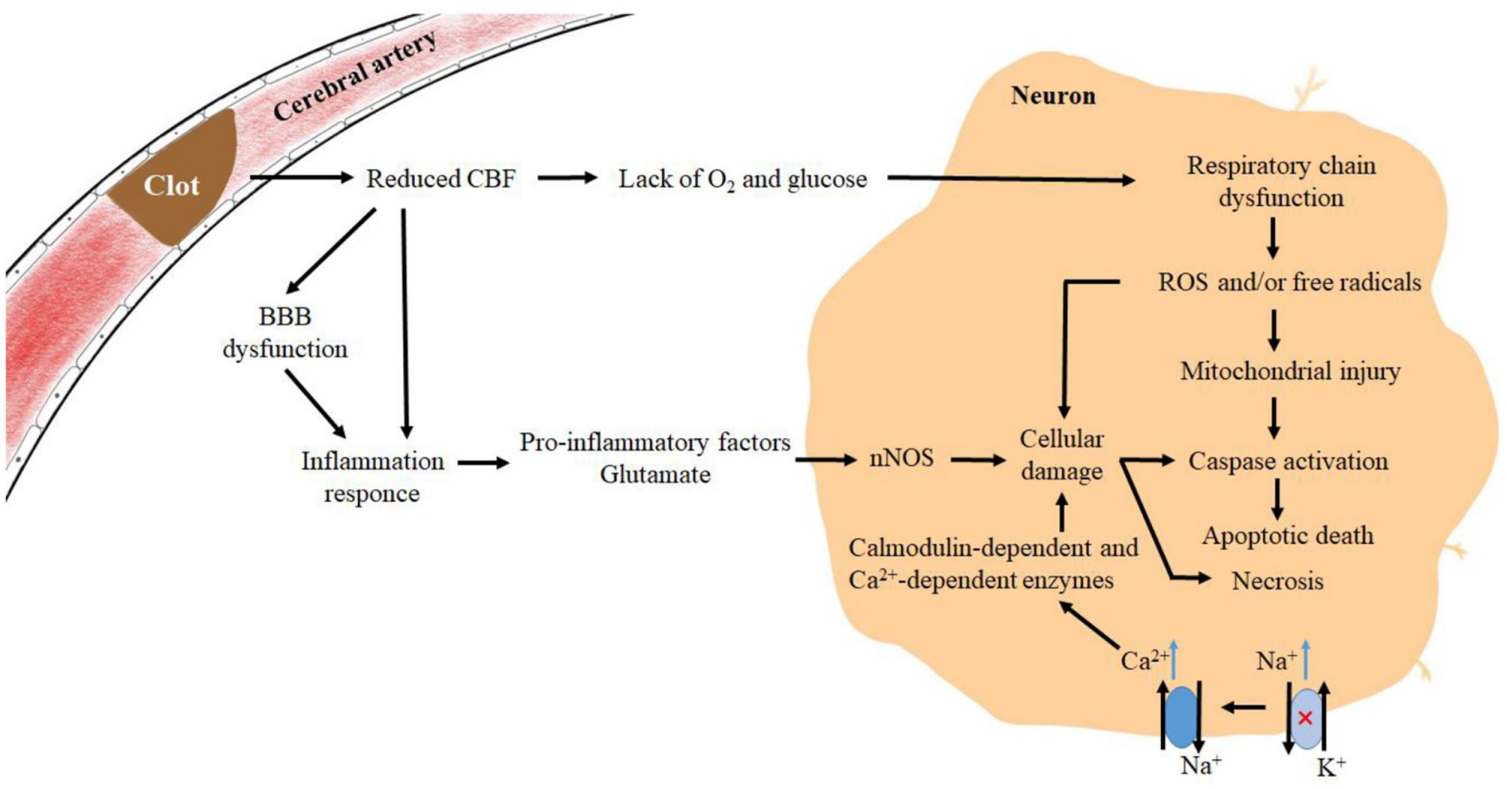
Mechanisms of pathophysiological changes in brain tissue after the onset of ischemic stroke. Pathophysiological changes in the corresponding regions during ischemic stroke, where a decrease in CBF and lacks of O_2_ and glucose leads to a large increase in excitatory amino acids, which act on receptors on the cell membrane and produce “amino acid toxicity”. Glial cells produce signaling factors that synergize with ischemia-induced BBB deficits and pro-inflammatory factors produced by microglia as a result of excitatory amino acids, exacerbating the inflammatory response and accelerating an increase of free radicals in neuronal cells, ultimately leading to cell death.

Stroke, classified as both ischemic stroke and hemorrhagic stroke, is responsible for major deaths and disabilities worldwide. Ischemic strokes account for 71% of strokes worldwide, and the current reperfusion strategies include intravenous thrombolysis and endovascular thrombectomy. Most ischemic strokes originate from thromboembolism, and a small proportion is caused by small vessel diseases (Campbell et al., 2019). When cerebral blood vessels become narrow and cerebral blood flow (CBF) declines for various reasons, the available glucose and oxygen will witness a decrease, resulting in a relative deficiency in energy production, disruption of the normal ion concentration gradient, and interference in normal membrane potential. Then, it is followed by the depolarization of the presynaptic membrane and an increase in excitatory amino acid transmitters (González-Nieto et al., 2020). In addition, the dysfunction of the Na^+^-K^+^ ATPase will elevate the intracellular Na^+^ concentration, thus further activating and reversing the Na^+^/Ca^2+^ exchanger. Under the action of excitatory amino acids, neurons release excitatory transmitters such as glutamate and dopamine. In the meanwhile, the intracellular Ca^2+^ will witness a rapid increase in glial cells and neurons, activating a variety of calmodulin-dependent and Ca^2+^-dependent enzymes that can disrupt cell structure and contribute to cell death (Abdullahi et al., 2018). Furthermore, the hyperexcitability of glutamate receptors drives neuronal production of NO synthases which is involved in the production of reactive oxygen species (ROS) (González-Nieto et al., 2020). When hypoxia occurs and the respiratory chain is in dysfunction, excess ROS and/or free radicals are generated in the cell, triggering a state of oxidative stress, which can damage the nucleic acids, proteins, and the structure of the cell membrane (Ayala et al., 2014). Furthermore, the dysfunction of the blood–brain barrier (BBB) and the release of signaling molecules from glial cells can promote inflammatory responses. Subsequently, excessive production of ROS and/or free radicals in neurons can cause structural damage and dysfunction of neuronal cells, predisposing the cells to apoptosis or necrosis (Campbell et al., 2019) (Figure 1). As a member of the SP family together with SP2, SP3, and SP4, SP1 serves as a crucial transcription factor and a multipotent oxidative stress response protein (Yeh et al., 2011). Participating in numerous downstream pathways, SP1 is known to be associated with 410 diseases including myocardial infarction, lipid metabolism disorders, and multiple cancers. It was found that 38 transcription factors were differentially expressed after transient middle cerebral artery occlusion (tMCAO) in mice, with SP1, SPi1, and Stat3 being the most significant (Rakers et al., 2019), and it has been hypothesized that SP1 and Argonaute 1 (AGO1) are the two major genes involved in ischemic stroke (Wei et al., 2020). Through facilitating the transcription of various antioxidant proteins, such as zinc finger protein 179 (Znf179), and various antioxidant enzymes, SP1 can affect the ion transporters on the cytoplasmic side to protect neurons, glial cells, and endothelial cells. However, there are still some mechanisms existing that play counterproductive roles and exacerbate the damage of cerebral ischemia. In this review, we have provided clear insight into the role of SP1 in ischemic stroke and further explored the therapeutic strategies associated with SP1 in ischemic stroke.

## Gene Structure, Composition, and Regulation of SP1

According to the GeneCards database, the *SP1* gene is located on the long arm of chromosome 12 at position 12q13.13. Human SP1 is composed of 785 amino acids with a total molecular weight of 80,693. Located in both the cytoplasm and nucleus of a cell, SP1 bears a high nuclear content and a low tissue specificity. It can be found in many organs such as the brain, kidney, pancreas, lymph, and bone marrow. Three zinc finger Cys2His2 structures, responsible for binding to GC-rich DNA sequences and enhancing gene transcription, make up the active centers of SP1.

The basic functions of SP1 include binding to basic helix-loop-helix (bHLH) transcription factors (Zheng et al., 2018), acting as a DNA transcription activator (Gilmour et al., 2019), combining with histone acetyltransferases/histone deacetylases (Li et al., 2019a), and interacting with HMG frame structural domains (Kovacevic Grujicic et al., 2005) (Table 1).

**Table 1:** Basic functions and physiological significance of SP1.

**Function**	**Physiological significance**	**Reference**
Binding to transcription factor, basic helix-loop-helix (bHLH)	Interacting non-covalently with the bHLH superfamily and serving as an important regulatory component of many developmental pathways	Zheng et al., 2018
DNA transcription activator	Binding to specific sequences of DNA activating or increasing specific gene sequences transcribed by RNA polymerase II	Gilmour et al., 2019
Binding to histone acetyltransferase/histone deacetylase	Increasing histone acetylation/deacetylation and activating/repressing transcriptional activities of genes	Li et al., 2019a
Interacting with the high-mobility group (HMG) domain or HMG box	Participating in the regulation of DNA-dependent processes (transcription, replication, strand repair)	Kovacevic Grujicic et al., 2005

The regulation of SP1 is mainly affected by posttranslational modifications such as phosphorylation, acetylation, ubiquitylation, sumoylation, glycosylation, and proteolytic lysis. There are also cap-independent pathways, namely, the internal ribosome entry sites (IRES) pathway, which eventually raise the translation efficiency by adding highly selective SP1 ribosomes (Yeh et al., 2011).

### Posttranslational Modifications of SP1

#### Phosphorylation Modification of SP1

Phosphorylation of SP1 can affect its transcriptional activity and stability. Currently, it is known that there are 61 phosphorylation sites of SP1, including 48 residues of serine, 10 residues of threonine, and 3 residues of tryptophan. Phosphorylation of SP1 will produce positive or negative regulation on DNA binding and transcriptional activity (Tan and Khachigian, 2009).

Phosphorylation of Sp1 at Ser101 depends on ATM, the major kinase responsible for Ser101 direct Sp1 phosphorylation. As DNA strand breaks accumulate, ATM phosphorylates transcription factor Sp1 and destabilizes it, which results in the downregulated transcription of the DNA base excision repair (BER) gene *XRCC1*, accompanied by decreased levels of DNA ligase III. Lower levels of both simultaneously increase the accumulation of DNA strand breaks, forming a vicious cycle, which is seen as a protective mechanism for removing unrepaired cells from permanent DNA strand breaks (Fletcher et al., 2018). The DNA binding ability and the transcription level of Sp1 change after the phosphorylation of Sp1 by different proteins (Tan and Khachigian, 2009).

#### Other Modifications of SP1

O-Linked N-acetylglucosamine (O-GlcNAc) modification of SP1, induced by hyperglycemia, mediates the expression of intracellular adhesion molecule 1, thus further promoting inflammation (Zhang et al., 2017). This modification may inhibit the transcriptional activation of glycolytic genes (Lim et al., 2015). In addition, the acetylation of SP1 can boost the growth of glioblastoma (Yang et al., 2020).

### Regulation of Translation Efficiency: The Cap-Independent Pathways

SP1 mRNA 5'-UTR contains a conserved sequence, the IRES, which is the translational regulatory element of mRNA first identified in viruses (Godet et al., 2019; Arhab et al., 2020). It forms a complex of high affinity with the ribosome subunit and dynamically manipulates its conformation to promote protein synthesis. The ability of these IRES to form high-affinity ribosomal complexes enables the production of structural and biochemical models for certain initiation steps and plays an important role in the structural dynamics of eukaryotic ribosomes (Arhab et al., 2020). Cap-containing IRES mRNA can be translated through either cap-dependent or IRES-dependent mechanisms. Studies have indicated that, under hypoxic conditions, there is a shift from cap-dependent pathways to cap-independent and IRES-dependent pathways (Bornes et al., 2007).

In a male rat model of oxygen and glucose deprivation (OGD) by using endothelin, large amounts of reactive oxygen species and H_2_O_2_ can elevate SP1. Typical IRES regions have been identified upstream of the SP1 coding region, which are activated by H_2_O_2_ to collect more ribosomes binding to mRNA, thus boosting the translation efficiency of SP1. In short, H_2_O_2_ improves the translation efficiency of SP1 by selectively increasing the number of ribosomes associated with SP1 mRNA (Yeh et al., 2011).

## The Role of SP1 in Ischemic Stroke

Ischemic stroke occurs when local blood flow to the brain is interrupted, followed by the hypoxia in nerve cells, glial cells, and endothelial cells in the ischemic region, where SP1 is upregulated and on the act. The role of SP1 in ischemic stroke can be divided into two different kinds, protective and damaging. The protective roles include its antioxidant, anti-apoptotic, anti-thrombotic effects, and its function in reducing brain edema through the formation of certain ion channels, while its damaging roles can be identified as a promoter of inflammation, a constricting factor of cerebral vessels, and an aggravator of brain edema through the formation of certain ion channels that serve as a double-edged sword.

### Antioxidant Effect of SP1

When nerve cells, glial cells, and endothelial cells are in an ischemic state and the respiratory chain is dysfunctional, excess reactive oxygen species and/or free radicals are generated intracellularly, putting the cells under oxidative stress. Biofilm structure is disrupted and lipid peroxidation occurs, producing toxic aldehyde products such as malondialdehyde (MDA) and 4-hydroxynonenal (4-HNE). Susceptible to these aldehyde modifications, the proteins and DNA are predisposed to functional and structural disorders. Moreover, MDA and hypoxia-inducible factor-1α (HIF-1α) can upregulate *SP1* gene expression. Antioxidant enzymes such as glutathione peroxidase (GPX), peroxidase (Prx), and superoxide dismutase (SOD) can inhibit oxidative damage. With the help of the reducing agent glutathione (GSH), GPX catalyzes the reduction of H_2_O_2_ or organic hydroperoxides into water or the corresponding alcohols (Ayala et al., 2014). SP1 attenuates the cellular oxidative stress state by directly or indirectly raising up the level of antioxidant enzymes.

Zinc finger protein 179, also known as RING finger protein 112 (Rnf112), is one of the most essential factors in the differentiation and development of the nervous system during embryogenesis, mainly expressed in the central nervous system. It serves as an important neuroprotector in ischemic stroke, neurodegenerative disease, and traumatic brain injury (Su et al., 2016; Chuang et al., 2017; Lee et al., 2018). In a neuron-like cell model (differentiated mouse neuroblastoma N2a cells), Znf179 was found to protect neurons against ROS by increasing the levels of peroxidase 3 (Prx3) and superoxide dismutase 2 (SOD2). In addition, Znf179 attenuates apoptosis and TNF-α production under H_2_O_2_ exposure (Su et al., 2016). Apart from that, peroxide damage boosts the promoter activity of Znf179. In the Znf179 promoter region, there are at least seven conserved SP1-binding elements that promote Znf179 transcription through their bindings to SP1. Furthermore, experiments conducted with green fluorescent protein (GFP)-Znf179-expressing cells have confirmed that the increase of Znf179 could induce the expression of its own promoter, forming a positive feedback loop (Chuang et al., 2017). Nerve growth factor (NGF) enhances the neuroprotective function of the SP1-Znf179 pathway by elevating the level of SP1 phosphorylation via the phosphatidylinositol 3-kinase/PKC-ζ pathway (Chuang et al., 2017). To conclude, mediated by SP1 and promoted by NGF, the activation of Znf179 autoregulatory loop matters a lot in reducing H_2_O_2_-induced oxidative stress toxicity in cells (Chuang et al., 2017). SAHA, a histone deacetylase (HDAC) inhibitor, was shown to promote the dissociation of Znf179-HDAC1 and the acetylation of the *Znf179* gene in differentiated N2a cells, which could enhance neuroprotection. Furthermore, SAHA could recruit SP1 to the Znf179 promoter to form the Znf179-SP1 complex and activate the transcription of Znf179 (Wu et al., 2018).

Peroxiredoxin 6 (Prdx6) is a bifunctional protein with glutathione peroxidase and Ca^2+^-independent phospholipase A2 (aiPLA2) activities (Fisher, 2011). A study identifies another antioxidant mechanism in neurons, namely, the SP1/Prdx6 pathway. The binding of SP1 to each of the three SP1-binding sites in the Prdx6 promoter can upregulate Prdx6 expression (Jia et al., 2017). However, there is a lack of reports on the role of aiPLA2 activity of Prdx6 in ischemic stroke. Additionally, SP1 binds to glutathione peroxidase 4 (GPX4) and the phospholipid hydrogen glutathione peroxidase (phGPx) promoter to regulate their expression and acts as an antioxidant (Dai et al., 2020).

TP53-induced glycolysis and apoptosis regulator (TIGAR) is a novel TP53-inducible protein involved in regulating both metabolic and neuroprotective pathways. Because of its structural similarity to phosphofructokinase-1 (FPK-1) and fructose-2,6-bisphosphate kinase in the glycolytic pathway, it could inhibit glycolysis and divert metabolites to the pentose phosphate pathway (PPP) by elevating glucose-6-phosphate dehydrogenase (G-6-PD) level (Lee et al., 2015; Chen et al., 2018). Experiments have shown that SP1 binds to the TIGAR promoter to promote transcription (Zou et al., 2012). Under ischemia and OGD/reoxygenation, SP1 has a significant role_in inducing TIGAR proteins in neurons and astrocytes, which has been confirmed in *in vivo* experiments in mice (Sun et al., 2015; Chen et al., 2018). Moreover, in astrocytes and neurons, SP1-induced TIGAR can counteract ROS and thus protect neurons and astrocytes by inhibiting NF-κB, reducing inflammatory factor levels, and increasing NADPH and GSH brought about by the PPP pathway, thereby reducing brain edema and shrinking the size of brain infarcts (Zhou et al., 2016; Chen et al., 2018; Duan et al., 2018).

### Anti-apoptosis Effect of SP1

Each member of the inhibitors of apoptosis proteins (IAPs) contains at least one baculovirus inhibitor of apoptosis repeat (BIR) that prevents programmed cell death. Survivin is the smallest molecular member of the IAPs involved in cell cycle progression and apoptosis inhibition, and normally, it is expressed only in proliferatively active cells and is overexpressed in most human cancers (Wheatley and Altieri, 2019). During the onset of ischemic stroke, the level of survivin in vascular endothelial cells undergoes an upregulation. Upstream factors, such as Smac released from mitochondria, inhibit IAPs by binding to the BIR structural domain, and there is evidence that survivin compromises apoptosis by suppressing caspase activator Smac. In addition, survivin also directly binds to and inhibits caspase-3 (Mansour et al., 2012). Through the above pathways, survivin alleviates the risk of cerebral hemorrhage after ischemic stroke by mitigating the destruction of the BBB triggered by apoptosis (Mallolas et al., 2014). The promoter of the *survivin* gene has a typical CpG island and many SP1-binding sites, making it possible for SP1 to activate and upregulate survivin transcription, which can be found in various studies such as ovarian cancer (Mak et al., 2017; Wang et al., 2017).

### Preventing DNA Damage by Inducing Cox-2 Expression

**Figure 2:**
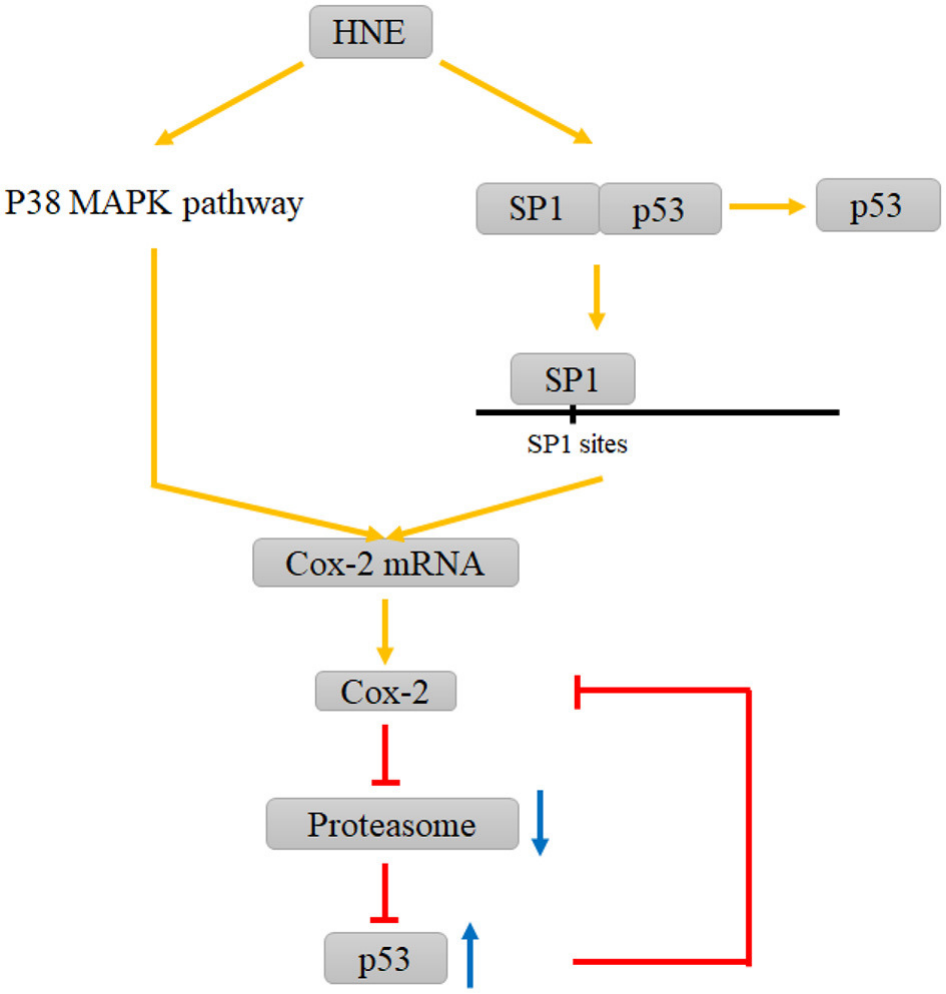
Interactions between HNE and COX-2, proteasome and P53 involving SP1. HNE elevates Cox-2 levels by promoting P38 MAPK pathway and the dissociation of the SP1-p53 complex. The increase in Cox-2 leads to the dysfunction of proteasomes and the accumulation of p53, which can inhibit this increasing trend via a negative feedback loop.

Under the ischemia-induced oxidative stress state, the phosphorylation of SP1 witnesses an upregulation. Then, SP1 promotes Cox-2 expression by binding to two SP1-binding sites proximal to the Cox-2 promoter. Either SP1 activity inhibition or Cox-2 deficiency exacerbates DNA damage in neurons (Lee et al., 2006). However, Cox-2 has also shown a pro-apoptotic effect in other diseases and nonneural cell studies (An et al., 2014; Song et al., 2019). As mentioned earlier, 4-HNE is an electrophilic lipid peroxidation product that can dysfunction the target molecules such as proteins and nucleotides possible to further induce apoptosis (Sonowal and Ramana, 2019). It can not only stabilize Cox-2 mRNA via the p38 mitogen-activated protein kinase signaling pathway but also facilitate the dissociation of the SP1-p53 heterodimer to produce SP1 accompanied by SP1 nuclear translocation after binding to the Cox-2 promoter. Relevant studies have demonstrated that Cox-2 is adversely regulated by p53, and the possible mechanism underlying this process may be attributed to the downregulation role of the formation of the SP1-p53 complex on functional SP1. Also, the upregulated level of Cox-2 is followed by the dysfunction of proteasomes and the accumulation of p53 and ubiquitinated proteins (Kumagai et al., 2015) (Figure 2). Therefore, the role of Cox-2, which is upregulated by SP1, in the oxidative stress state of neurons and glial cells during the onset of ischemic stroke needs to be further explored.

### Damaging and Protective Effects of SP1 *via* HIF-1

Hypoxia-inducible factor-1α (HIF-1α), mostly expressed in microglias and neurons during the onset of ischemic stroke, is a transcription factor that regulates oxygen levels. Its promoter involves the SP1 binding sites, suggesting the potential role of SP1 to induce *HIF-1* gene transcription under hypoxia (Rhim et al., 2013). In astrocytes, matrix metalloproteinase-2 (MMP-2), activated via a hypoxia-inducible factor-1α (HIF-1α)-dependent mechanism, affects the vascular nerve unit (VNU) and eventually degrades the tight junctions of the BBB and induces BBB breakdown (Abdullahi et al., 2018). In addition, HIF-1α may promote an inflammatory response through the NLRP3 inflammasome after stroke (Jiang et al., 2020). Besides, SP1 can co-localize with HIF-1 and histone acetyltransferase p300 in the Na^+^/Ca^2+^ exchanger 1 (NCX1) gene sequence, causing epigenetic changes and upregulation of NCX1 expression, producing neuronal protective effects, which will be further discussed in the following Section 3.5 (Formisano et al., 2015).

### SP1 and Ion Transporters on the Cytosolic Membrane

Na^+^/Ca^2+^ exchanger 1 has been proved to be crucial in attenuating brain injury after cerebral ischemia (Valsecchi et al., 2011; Chuang et al., 2017). NCX regulates both the intracellular and extracellular contents of Na^+^ and Ca^2+^ according to membrane potential and transmembrane ion gradients (Shenoda, 2015). It was found that by transient middle cerebral artery occlusion (tMCAO) in rats, SP1 and HIF-1 co-localized with histone acetyltransferase p300 on NCX1-brain promoter in cortical neurons and SP1/HIF-1/p300 resulted in high acetylation of NCX1-brain histone H3. Moreover, SP1 is a transcriptional activator of NCX1. The overexpression of NCX1 can counteract the death-promoting effect of p300 inhibitors on neurons under OGD/R condition. It is evident that SP1 ultimately shows a neuronal protective effect through NCX1 overexpression and epigenetic alterations caused by p300 (Formisano et al., 2015). It is worth noting that the development of drugs that modulate NCX1 by promoting its upregulation in stroke or through epigenetic changes can provide a novel and promising pharmacological way to ameliorate neuronal injury during cerebral ischemia.

However, SP1 does not just simply exert a protective effect on neurons and glial cells under ischemia or OGD/R, as there has evidence that it can also exacerbate the damage caused by ischemia.

Sulfonylurea receptor 1 (SUR1) is a member of the ATP-binding cassette (ABC) transporter protein family, and SUR1-NC_Ca_-ATP channels, also called SUR1-TRPM4 heterodimer, are almost absent in normal brain. SUR1-TRPM4 channels are composed of the Ca^2+^-activated nonselective ion channel TRPM4 and the regulatory subunit SUR1 (Mehta et al., 2015). In the state of oxidative stress caused by ischemia, SP1 and HIF-1 bind to the promoter of the *Abcc8* gene encoding SUR1 (Simard et al., 2012; Woo et al., 2012). In astrocytes and microvascular endothelial cells, the upregulation of SUR1 regulates TRPM4 for nonselective monovalent cation endocytosis. The heterologous complex SUR1-TRPM4-AQP4, formed by combining with AQP4, can enhance the permeability to water and ions, thus leading to the edema of astrocytes and enlarged infarct size (Simard et al., 2012; Stokum et al., 2018).

### Exacerbating Glutamate Excitotoxicity by Affecting t-PA

Tissue-type plasminogen activator (t-PA) is a fibrinogen activator that converts fibrinolytic enzymogen into fibrinolytic enzymes, triggering fibrinolysis and promoting clot lysis. By far, t-PA has been the only choice for thrombolytic therapy in acute ischemic stroke, but recent studies have shown that it has far-reaching effects on many other systems beyond the hematologic system, such as the immune system, the nervous system, and so forth (Draxler et al., 2019). In the brain, t-PA is produced by neurons, microglia, endothelial cells, and astrocytes, whose low-level expression can be witnessed in synaptic remodeling processes under physiological conditions.

In neurons and glial cells, enhanced t-PA levels during cerebral ischemia can influence the size of the infarct from ischemic stroke. The *t-PA* gene promoter can be regulated by many hormones, such as steroid hormones, to increase transcriptional activity under the induction of SP1. The ubiquitous character of polymorphisms of *t-PA* gene sequences is associated with the release of t-PA. The abundance of the wild-type C allele is approximately 30% more than that of the mutant T allele, and the t-PA enhancer-7351C > T SNP diminishes the affinity of SP1 for this locus, a change that reduces the release of t-PA and thus affects the final infarct size in cerebral ischemia (Tjarnlund-Wolf et al., 2011). It is worth noting that high levels of t-PA during ischemia may increase the excitotoxicity of glutamate through the cleavage and activation of NMDA receptors, ultimately aggravating the death of neurons and glial cells (Lopez-Atalaya et al., 2008). In the acute phase of ischemic stroke, neuronal and astrocyte t-PA secretion levels become the determinants of infarct size and long-term recovery. However, in the recovery phase of ischemic stroke, exogenous t-PA may increase the axon regeneration of the cerebral cortex through the epidermal growth factor receptor (EGFR) signaling pathway (Pu et al., 2019).

### Exacerbating Adverse Cerebral Vasoconstriction *via* ET_B_R Upregulation

It has been found that certain contractile G protein-coupled receptors are increased in brain tissue after ischemia-reperfusion (I/R) in human cerebral vessels (Edvinsson and Povlsen, 2011), among which the increases in endothelin type A (ET_A_R) and type B receptors (ET_B_R) are involved in the pathological process after stroke. Endothelin-1 (ET-1) plays a vasoconstrictive role in I/R, leading to a worsened prognosis. SP1, phosphorylated by ERK1/2, effectively upregulates ET_B_R expression in human cerebral arterial smooth muscle (Grell et al., 2014).

### Role of SP1 in Cerebral Hemorrhage

In the region of cortical contusion, SUR1 has also witnessed a significant upregulation in the cerebral cortex, microvasculature, and neurons. Thereafter, the formation of SUR1-TRPM4 channels can swell vascular endothelial cells, whose death could disrupt the vascular integrity and aggravate ischemia. By blocking the microvascular *Abcc8* gene, secondary hemorrhage after brain contusion can be reduced (Simard et al., 2009). Similarly, hemorrhagic necrosis occurring from spinal cord injury is transiently suppressed by inhibiting SUR1 expression (Simard et al., 2010). SUR1 is also upregulated in microvascular endothelial cells when an ischemic stroke occurs. The specific results are not elaborated, but the outcome may be similar to that of SUR1 upregulation after brain contusion.

SP1 and transcription factor AP-2 (TFAP2C) could upregulate glutathione peroxidase 4 (GPX4) expression (Dai et al., 2020). Studies have shown that pharmacological selenium or Tat SelPep (a selenoprotein that transports selenium intracellularly) significantly enhances the binding of TFAP2C and SP1 to *GPX4* gene upstream, increasing GPX4 expression and protecting neurons from hemin-induced iron death. It has also been demonstrated that inhibition of SP1 activity has almost completely abolished the role of Tat SelPep in the model of cerebral hemorrhage, indicating the central role of SP1 in the antioxidant processes in neuronal cells regulated by selenium (Alim et al., 2019). Selenium also seems to play an important neuroprotective role in ischemic stroke. By targeting selenium nanoparticles in the ischemic region of the central nervous system, it can modulate cellular metabolism, anti-oxidation, anti-inflammation, neuron repair, and many other signaling pathways, which play a crucial role in resisting ischemic stroke (Amani et al., 2019). Analogous to hemorrhagic stroke, the upregulation of SP1 may enhance the effect of selenium in ischemic stroke.

## Therapeutic Strategies Based on SP1 and Future Prospects

According to the summary about the role of SP1 in the development of ischemic stroke in this review, it is through the upregulation of SP1 level and the reduction of its degradation that the protective function of SP1 takes effect. As mentioned above, the promoter of the *survivin* gene has many SP1-binding sites, and it has been shown that Tanshinone IIA may exert neuroprotective effects after ischemia through the activation of the SP1/survivin pathway (Tang et al., 2019). In addition, SP1 promotes NCX1 overexpression and attenuates brain edema, and the development of drugs that modulate NCX1 by promoting its upregulation in stroke or through epigenetic changes may be a novel pharmacological pathway to improve neuronal injury during cerebral ischemia. Quite importantly, SP1 plays an antioxidant role during ischemic stroke. Through the activation of the endogenous *SP1* gene or the reduction of SP1 degradation, it is possible to promote its role as an antioxidant and attenuate the damage caused by ischemia. Curcumin exerts its antioxidant effect during I/R injury by activating SP1-dependent Prdx6 expression (Jia et al., 2017). Many studies have suggested that, in the near future, various novel drugs and therapies can be developed to elevate the intracellular level of SP1. In the study of renal vascular I/R, investigators innovatively used human-induced pluripotent stem cell-derived mesenchymal stromal cell-extracellular vesicles (hiPSC-MSCs-EV) to target SP1 delivery to protect the kidney from I/R injury (Yuan et al., 2017). Apart from that, it has been experimentally demonstrated that the upregulation of SP1 is accompanied by the reduction of myocardial and intestinal I/R injury, embodying its protective role effectively (Li et al., 2019b; Hu et al., 2020).

Conversely, taking into account its deteriorating effects in ischemic stroke, it is necessary to reduce the SP1 level or disturb and attenuate its downstream results. SP1 promotes the formation of SUR1-NC_Ca_-ATP channels, a complex that exacerbates brain edema. Glibenclamide targeting of SUR1 provides a therapeutic approach to prevent and treat brain swelling in ischemic stroke (Woo et al., 2020). Interfering with the binding activity of SP1 to the *Abcc8* gene promoter is one of the existing ways to improve cellular edema in a state of oxidative stress in the brain, and resveratrol has been shown to have this effect (Alquisiras-Burgos et al., 2020). Some strong antioxidants such as polyphenolic compounds like CUR, glutathione byproducts like NAC, affect the expression of SUR1 in oxidative stress by dampening the activity of SP1 and NF-κB/p65 transcription factors (Chatterjee et al., 2019). Given that SP1 elevates ETBR levels and exacerbates cerebral ischemia, relevant studies have confirmed that mithramycin A can be used to reduce ETBR expression on cerebral arterial smooth muscle after I/R (Grell et al., 2014).

Interestingly, the levels of certain miRNAs are altered during cerebral ischemia, and SP1 can serve as a target of these miRNAs related to stroke. Hence, further epigenetic researches hold a quite promising future for stroke treatment. When the proinflammatory role of SP1 in ischemic stroke is confirmed, various treatments aimed at its reduction can be learned from similar methods used in the cures of other diseases. With the evidence showing its association with tumorigenesis, SP1 also gets actively involved in several cancers (Beishline and Azizkhan-Clifford, 2015). In ovarian cancer studies, SP1 has been found to regulate genes overexpressed in the cancer process, and the direct targeting of it by miR-128 and miR-377 can retard the proliferation of cancer cells (Chen et al., 2019). Various treatments and drugs targeting SP1 have been investigated to cure cancers. For example, mithramycin A and its analogs can block the binding of SP1 to the gene and further inhibit its function as a transcription factor in multiple ways (Previdi et al., 2010; Sankpal et al., 2011).

In addition, many studies have proved the proinflammatory effects of SP1, which are quite common in neurodegenerative diseases. An increase in SP1 is also associated with inflammatory responses in degenerative diseases of the central nervous system. SP1, a proinflammatory factor, is significantly elevated in the brain in patients with Alzheimer's disease or Parkinson's disease. It has been found that upregulation of miR-375 attenuates dopaminergic neuronal damage in Parkinson's disease by inhibiting SP1 and attenuating oxidative stress and inflammatory responses (Cai et al., 2020). Moreover, during spinal cord I/R injury, miRNA-128-3p exerted a neuroprotective and inflammation-reducing effect by inhibiting SP1 and reducing the proinflammatory factors IL-6, TNF-α, and IL-1β (Wang et al., 2020). The important role of SP1 in upregulating inflammatory factors indicated in the studies mentioned above also implies that through the inhibition of SP1, the reduction of inflammatory factors and cell protection can be achieved. Although many studies and reports on SP1 affecting inflammatory factors in neurodegenerative diseases are currently available, the relationship between SP1 and inflammatory factors in ischemic stroke still needs further investigation.

**Figure 3:**
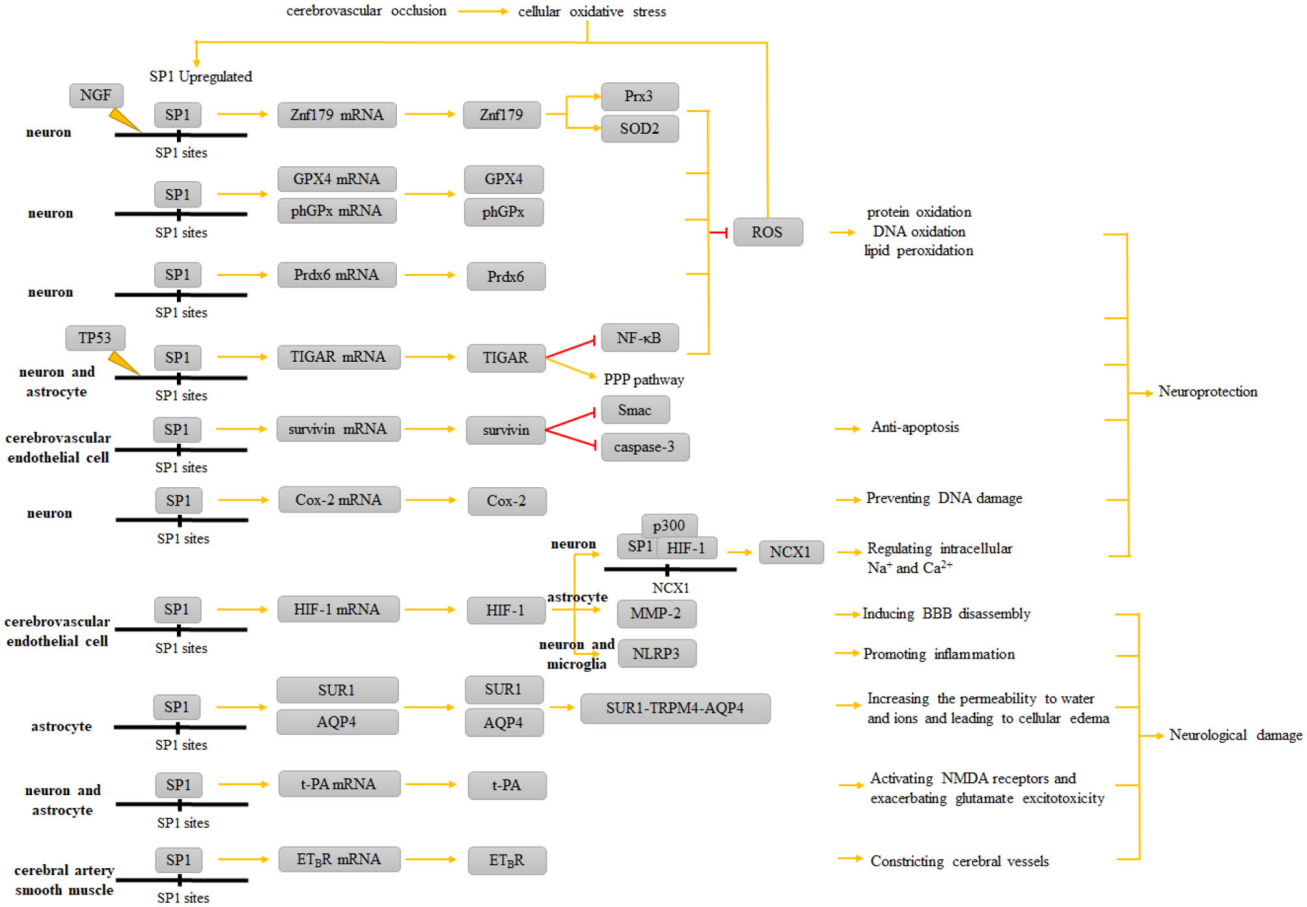
Role of SP1 in ischemic stroke. When inflammatory responses occur in the corresponding ischemic areas of the brain, increased intracellular SP1 upregulates the expression of Znf179, GPX4, phGPx, Prdx6, and TIGAR, which ultimately resist ROS and ameliorate protein oxidation, structural DNA oxidation and lipid peroxidation; upregulated survivin resists apoptosis; Cox-2 prevents DNA damage; HIF-1 can cooperate with SP1 to form the SP1-p300-HIF-1 complex, which upregulates NCX1 and protects neurons. However, the neurological damage caused by SP1 is reflected in MMP-2 and NLRP3 synthesis induced by the upregulation of HIF-1; upregulated SUR1 and AQP4 exacerbate brain edema; t-PA exacerbates glutamate excitotoxicity; ETBR enables the vasoconstrictive effect of ET-1.

SP1 is a common DNA-binding protein involved in a variety of physiological and pathophysiological processes. In this review, we present a comprehensive overview on the roles of SP1 as both an antioxidant and anti-apoptotic agent, as well as how it affects the cytosolic ion transporters and promotes inflammation in ischemic stroke events. It is not difficult to find that SP1 has both protective and non-protective effects on neurons and neuroglial cells (Figure 3 and Table 2). Further studies on the role of SP1 in ischemic stroke should focus on clarifying the periods and stages when SP1 exerts different effects, the situations where its effects tend to be protective or damaging, and the factors that influence the manifestations of its diverse effects. In the future, the role of SP1 in ischemic stroke and related treatment needs detailed investigation to amplify its protective role while dampening its damaging effects.

**Table 2:** Roles and significance of SP1 in ischemic stroke.

**Function**		**Cell type**	**Physiological significance**	**Reference**
Protective effects	Antioxidant	Neuron and astrocyte	SP1 binds to the promoters of *Znf179, GPX4, Prdx6*, and *TIGAR* genes to increase the expression of the corresponding genes, ultimately protecting neurons through antioxidant effects.	Zou et al., 2012; Su et al., 2016; Zhou et al., 2016; Chuang et al., 2017; Jia et al., 2017; Chen et al., 2018; Wu et al., 2018
	Anti-apoptosis	Cerebrovascular endothelial cell	SP1 activates survivin transcription and exerts neuroprotective effects through the SP1/survivin pathway.	Mallolas et al., 2014
	Preventing DNA damage	Neuron	SP1 binds to the promoter of *Cox-2* and promotes Cox-2 expression, preventing DNA damage.	Lee et al., 2006
	Promoting Na^+^/Ca^2+^ transporter 1 expression	Neuron	SP1 and HIF-1 together with histone acetyltransferase p300 are localized to NCX1 promoter, and NCX1 overexpression counteracts p300 inhibition-induced neural death.	Formisano et al., 2015; Shenoda, 2015
Damaging effects	Inducing BBB catabolism and inflammatory response	Astrocyte (for BBB catabolism) and neuron and microglia (for inflammatory response)	SP1 induces *HIF-1* gene transcription under hypoxia. MMP-2 activated by HIF-1α-dependent mechanism can induce BBB catabolism, while HIF-1α regulates the inflammatory response through the NLRP3 inflammasome complex.	Abdullahi et al., 2018; Jiang et al., 2020
	Leading to cerebral edema	Astrocyte	SP1 promotes the formation of SUR1-TRPM4-AQP4 complex that increases permeability to water and ions, leading to cellular edema.	Simard et al., 2012; Woo et al., 2012; Stokum et al., 2018
	Exacerbating glutamate excitotoxicity	Neuron and astrocyte	SP1 binds to the enhancer of t-PA, promoting the release of t-PA which activates NMDA receptors and exacerbating glutamate excitotoxicity.	Lopez-Atalaya et al., 2008
	Constricting cerebral vessels	Cerebral artery smooth muscle	SP1 upregulates ET_B_R expression, which enables ET-1 to exert a vasoconstrictive effect.	Grell et al., 2014

## Author Contributions

MZ conceptualized the study, acquired funding, and administered the project. QY, MZ, and WL wrote the original draft. ZC provided the resources. MZ, QY, and ZC reviewed and edited the manuscript. All authors contributed to the article and approved the submitted version.

## Funding

This research was funded by the Natural Science Foundations for Excellent Young Scholars of Hunan Province (No. 2021JJ20095), the Key Research and Development Program of Hunan Province (No. 2020SK2063), Research Project on Education and Teaching Innovation of Central South University (2021jy145), the Natural Science Foundations of Hunan Province (No. 2020JJ4134), and the National Natural Science Foundation of China (No. 81501025).

## Conflict of Interest

The authors declare that the research was conducted in the absence of any commercial or financial relationships that could be construed as a potential conflict of interest.

## Publisher's Note

All claims expressed in this article are solely those of the authors and do not necessarily represent those of their affiliated organizations, or those of the publisher, the editors and the reviewers. Any product that may be evaluated in this article, or claim that may be made by its manufacturer, is not guaranteed or endorsed by the publisher.
